# Compliance, illiteracy and low-protein diet: multiple challenges in CKD and a case of self-empowerment

**DOI:** 10.1186/s12882-016-0353-0

**Published:** 2016-09-29

**Authors:** Stefania Maxia, Valentina Loi, Irene Capizzi, Giorgina Barbara Piccoli, Gianfranca Cabiddu, Antonello Pani

**Affiliations:** 1 SC Nephrology and Dialysis, Brotzu Hospital, Piazzale Alessandro Ricchi 1, 09134 Cagliari, Italy; 2 SS Nephrology, SCDU Urologia, Department of Clinical and Biological Sciences, University of Torino, San Luigi Gonzaga Hospital, Regione Gonzole 10, 10043 Orbassano, Italy; 3 Nephrologie, Centre Hospitalier Le Mans, 194 av. Rubillard, 72037 Le Mans, France

**Keywords:** Case report, Illiteracy, Low protein diet, Chronic kidney disease, Compliance

## Abstract

**Background:**

Low-protein diets (LPD) are an important means of delaying the need for dialysis and attaining a stable metabolic balance in chronic kidney disease (CKD). Many authors consider a low educational level and illiteracy to be adverse features for a good dietary compliance.

**Case presentation:**

We report the case of a 77-year old woman, illiterate, affected by advanced CKD (stage 4 according to KDIGO guidelines). She was initially ashamed of her problem and did not declare it, leading to an overzealous reduction in protein intake. However, with her daughter’s help, who translated the dietary prescription into images, she overcame the barrier represented by illiteracy and was able to correctly follow the prescriptions, attaining good kidney function stability and preserving an adequate nutritional status.

**Conclusions:**

The case underlines the importance of a personalized approach to dietary prescriptions and suggests that it is possible to achieve a good compliance to the dietary treatment of CKD also in patients with relevant cultural barriers.

## Background

Low protein diets are considered effective tools in reducing proteinuria, correcting and preventing signs, symptoms, and complications of chronic kidney disease (CKD), delaying the start of dialysis, preventing malnutrition and providing cardiovascular protection [1].

The use of low-protein diets is still open for debate. Besides the risk of malnutrition, the main reason why many authors feel diets are not worth prescribing is that it is often met with poor compliance, especially when the diet is combined with the complex therapies that are usually needed in our patients with advanced CKD [2–6].

Adherence to the prescriptions is also linked to the educational level; however, recent studies have underlined that the educational level may not be an absolute barrier in motivating patients and attaining compliance [7, 8]. While patients usually prefer direct counselling with the caregivers, visual aids may be useful in improving compliance [9]. Their potential limitation may be the lack of correspondence to a shared language, particularly in patients with a low educational level [9–11].

Illiteracy, justly considered a “silent epidemic”, is not negligible in several areas, including developed countries, especially in elderly patients. The importance of illiteracy was underlined in a recent case report published in *New England Journal of Medicine*, showing how low compliance to anti-diabetic drugs was resolved by the “diagnosis” of illiteracy [12].

Our case report describes an illiterate patient with severe CKD, who created with her daughter a clear and simple visual aid allowing good compliance; her story calls once more for attention to this neglected social and clinical problem, and conversely suggests that illiterate patients may provide important lessons on compliance and empowerment. While warning against discrimination of patients with a low educational level, this case underlines the importance of taking time in the clinical practice to consider cultural barriers which could potentially impair the success of the care in CKD patients.

### Case presentation

A 77 years old woman was referred in 2014 to our outpatient unit dedicated to advanced CKD in the Brotzu Hospital in Cagliari, Sardini, from a different nephrology unit where she had been followed since 2005. In 2006, a kidney biopsy led to the diagnosis of focal and segmental glomerulonephritis with advanced tubular interstitial damage.

Her clinical history was characterised by long-lasting hypertension (for at least 30 years). In 2011, she underwent total thyroidectomy, due to a multi-nodular colloid-cystic goitre. In 2013, she underwent a radical left mastectomy for a ductal infiltrating carcinoma (G2 pT2 pN3) and was treated afterwards with radiotherapy and aromatase inhibitor. Furthermore, in the past few years, she had lost a considerable number of teeth and suffered from chewing difficulties.

The patient had been referred to our unit because of a rapid worsening of her kidney function, with an estimated Glomerular Filtration Rate (eGFR), calculated with the Chronic Kidney Disease – Epidemiology Collaboration (CKD-EPI) formula that decreased from 25 to 16 ml/min in five months. An in-depth history and a basic workout had ruled out the most common causes of rapid worsening of the renal function, including dehydration caused by infectious illness or by climatic conditions, ingestion of NSAIDs or any other drug out of those prescribed, cardiac, and vascular disease. At referral, her therapy included levothyroxine 100 mcg, furosemide 50 mg, losartan 50 mg, lercarnidipine 10 mg, ramipril 10 mg, allopurinol 150 mg, ezetimibe 10 mg/simvastatin 20 mg, calcium carbonate 1.25 g twice a day and cholecalciferol 25,000 UI every other week.

The patient lived with her husband and had had four children; a son and a daughter lived close by. One son had been on hemodialysis and had died at the age of 48 years from sepsis.

The patient, living in the countryside, was illiterate, a rare but not exceptional situation in her age group in our region [13].

At the first physical evaluation, the patient was overweight (67 kg, 154 cm, BMI 28.3 kg/m^2^) and the blood pressure control was suboptimal (PA 150/90 mmHg without difference in orthostatism).

The main biochemical data and the treatments are reported in Tables 1 and 2. Of note, she was on an association of angiotensin converting enzyme inhibitors (ACEi) and angiotensin receptor blockers (ARBs), which is employed in our setting in patients with nephrotic syndrome, and which was continued, in the absence of hyperkalemia at circa-monthly blood tests, also on account of the anamnestic data of a sharp increase in proteinuria if one of the two drugs was discontinued.

**Table 1:** Clinical and laboratory parameters

		Pre diet^a^	Pre visual aid (after diet start)	Post visual aid (1 year later)
Body weight	(Kg)	68	60,500	60
Creatinine	(mg/dL)	2.35	3.79	2.61
eGFR CKD-EPI	(mL/min)	19	11	17
Bun	(mg/dL)	67	84	76
Sodium	(mEq/L)	139	137	141
Potassium	(mEq/L)	4.4	4.9	4.6
Calcium	(mg/dL)	9.7	8.9	8.6
Phosphorus	(mg/dL)	4.4	4.8	4.3
Urine volume	(mL)	2700	1650	2300
Proteinuria	(g/day)	2.73	0.65	1
Hemoglobin	(g/dL)	11.4	9.9	9.4
Urinary urea	(g/day)	6.12	4.46	7.31
Mitch formula	(g/kg/day)	0.5	0.42	0.57
PH		7.359	7.367	7.390
Bicarbonate	(mmol/L)	34.2	30	29.3
Base excess	(mmol/L)	7.44	3.87	3.97
Total protein	(g/dL)	7.7	-	7.2
Albumin	(g/dL)	4.4	4.3	3.9
Glucose	(mg/dl)	90	89	77
Tot cholesterol	(mg/dL)	195	124	107
HDL Cholesterol	(mg/dL)	78	52	57
LDL Cholesterol	(mg/dL)	91	55	33
Triglycerides	(mg/dL)	129	87	84
Iron	(ug/dL)	76	48	46
Transferrin	(mg/dL)	129	147	189
Ferritin	(ng/mL)	339	325	280
Folic acid	(ng/mL)	6.3	6.6	>24
PTH	(pg/mL)	16	26	17
25- OH Vitamin D	(ng/mL)	32.5	38.6	32.5

*eGFR CKD-EPI* eGFR calculated by means of CKD EPI formula, *BUN* Blood Urea Nitrogen, *Mitch formula* Protein intake/Kg, according to the Maroni Mitch formula [30], *PTH* parathyroid hormone

^a^the patient had received some generic counselling and was avoiding virtually all animal derived proteins

**Table 2:** Therapy

	Pre diet*	Pre visual aid (after diet start)	Post visual aid (1 year later)
Levothyroxine	100 mcg	100 mcg for 6 days/week, 50 mcg for 1 day/week	100 mcg for 6 days/week
Furosemide	25 mg/twice a day	25 mg/twice a day	25 mg/twice a day
Losartan	50 mg	50 mg	50 mg
Lercarnidipine	10 mg	discontinued	/
Ramipril	10 mg	10 mg	10 mg
Allopurinol	150 mg	150 mg	150 mg
Ezetimibe/simvastatin	10 mg/20 mg	10 mg/20 mg	10 mg/20 mg
Calcium carbonate	1.25 g/twice a day	discontinued	/
Calcium acetate	/	500 mg/twice a day	500 mg/twice a day
Cholecalciferol	25,000 UI/every other week	discontinued	/
Anastrozole	1 mg	1 mg	1 mg

The dietary history revealed a relatively high protein intake (estimated as above 1 g/Kg/day on actual body weight by dietary recall) divided into three main meals and a midmorning snack, with a high consumption of pasta and bread. Since the importance of reduction in protein intake had been discussed in a previous clinical visit, at referral she had tried to self-manage her diet, resulting in an unbalanced low-protein diet, completely avoiding animal proteins and reducing the caloric intake. This overzealous attitude is a common and often underestimated problem in particular in elderly patients “scared” of dialysis. Almost paradoxically, in such cases, starting a “low protein diet” may lead to an increase in animal-derived proteins, to attain a stable balance, protective not only for the nutritional status but also for the renal function [14–18].

This was the case also in our patient. On account of the calculated previous protein intake, we attempted to re-equilibrate the diet by substituting the normal carbohydrates, on which the Italian diet is based, with protein-free food (notably available free of charge in Italy), with a target intake of 0.6 g/kg/day (based on her actual body weight, which roughly corresponded to a 0.6 g/Kg/day on ideal body weight), increasing at the same time the animal-derived proteins. The diet included a daily intake of 1.3 g of sodium, 2 g of potassium and 800 mg of phosphorus.

**Fig. 1:**
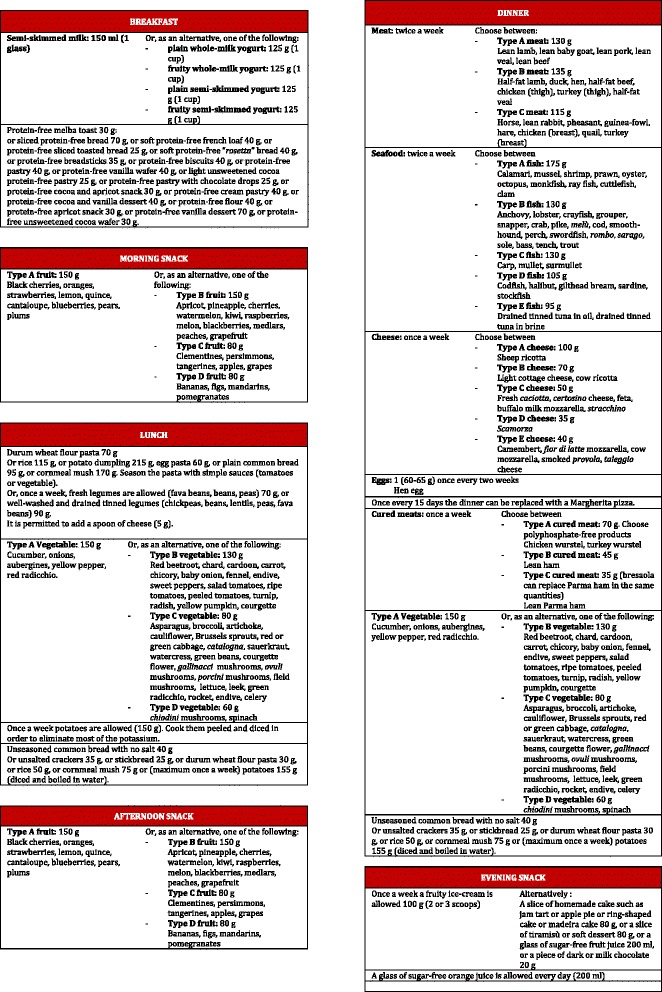
Written diet

At the first clinical visit after the diet prescription, kidney function was further reduced (Table 1), and the patient reported difficulties in following the prescribed diet (Fig. 1).

Notwithstanding her difficulties, the patient appeared motivated in following any advice that could postpone dialysis (mostly on account of having had a son on dialysis who had prematurely died), the family was involved in the counselling process and underwent extensive counselling during the following clinical visits.

**Fig. 2:**
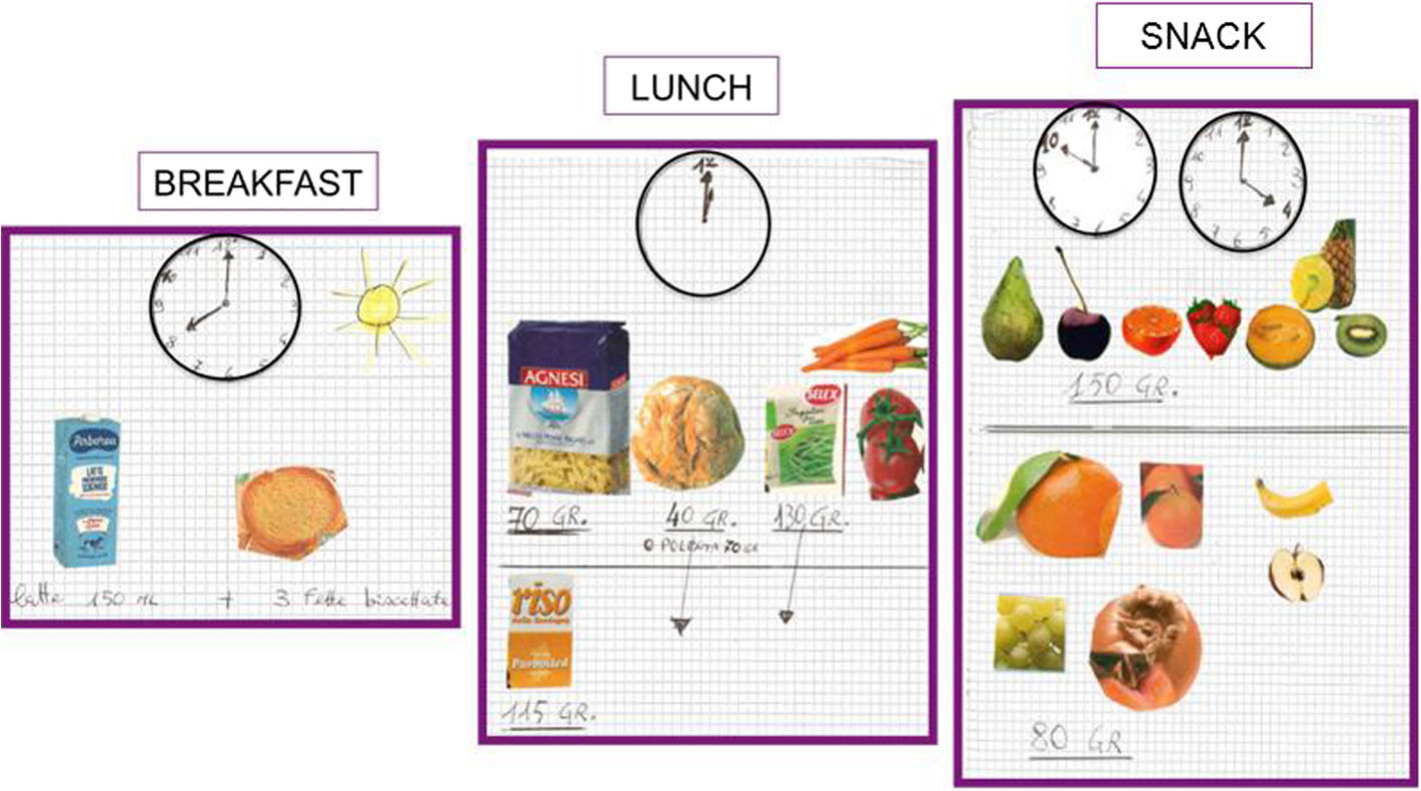
Visual aid system-diet [1]

**Fig. 3:**
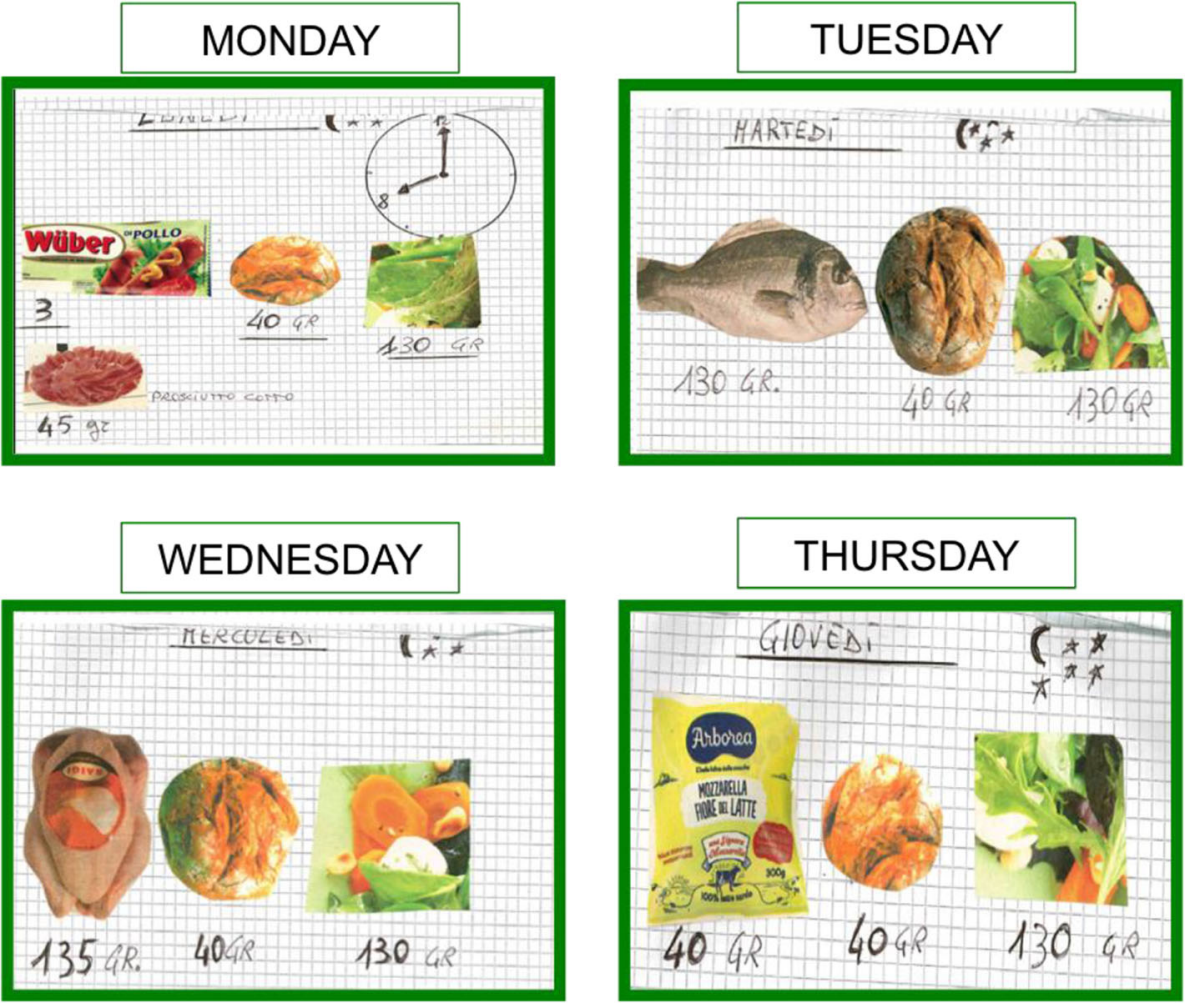
Visual aid system-diet [2]

**Fig. 4:**
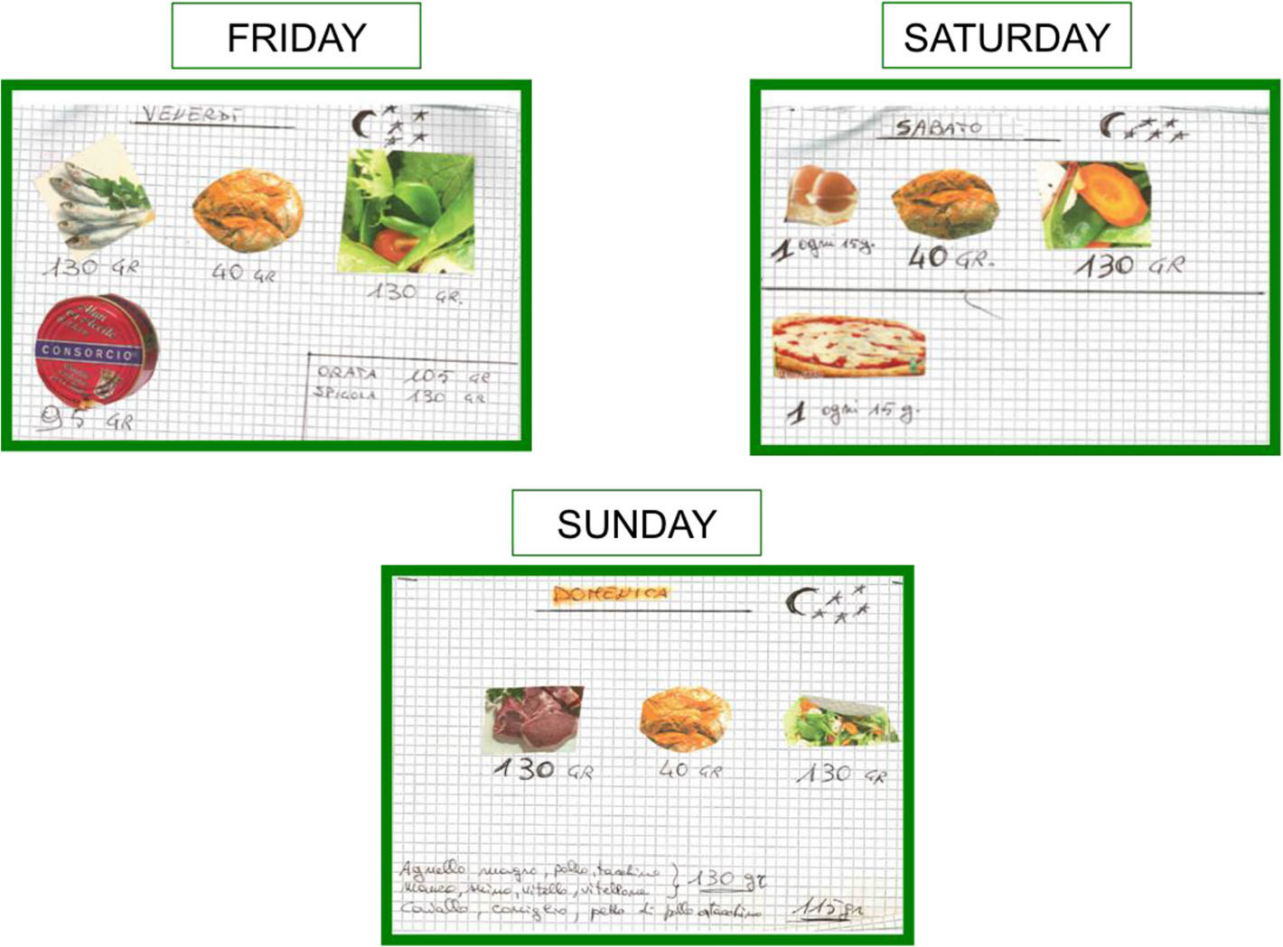
Visual aid system-diet [3]

At the following visit, overall compliance (including protein intake, caloric intake, and distribution of the food over the meals and food choice) was remarkably improved (Table 1). When asked how she had overcome her initial problems, the patient showed us the images reproduced in the Figs. 2, 3 and 4. Her daughter had built with her an extensive visual aid system, by translating the prescriptions into images taken from tabloids and advertisements, as shown. Indeed, it was only by this revelation that we discovered that our patient was illiterate, an issue that we had not taken into account, since she had hidden this information, being ashamed of her condition.

One and half years later, she was following the diet with good compliance, stable GFR and satisfactory nutritional status (Table 1). Indeed, this case made us reflect on more general terms on the difficulties in following complex diet plans, such as those proposed in our setting, and is leading us to shift towards a qualitative and simplified approach to low protein diets [19].

## Discussion

This clinical case shows how a low protein diet could also be followed where the premise does not look promising. Illiteracy is indeed an important obstacle as the impossibility to rely on written aids makes the daily management more difficult.

When prescribing a diet, the evaluation of the education level is not a point to be underrated, as even the best possible diet would never be successful if not understood by the patient. Our patient presented with this huge barrier: she was illiterate, and she was consequently unable to follow a written diet; moreover, being ashamed of her lack of education, she did not declare it in the beginning. This is not an exceptional problem in our setting: in Sardinia, in the 1940s, the rate of illiteracy was quite high, especially in the countryside and among women. Within a farmer’s family, children would generally start working from the earliest age possible and would not even attend primary school, which was the case for our patient [13].

When we realised this challenging problem, we decided not to give up because the patient, who had lost a son on haemodialysis, strongly refused the future option of renal replacement therapy.

A number of studies demonstrate an association between low educational level and low dietary compliance. Hadžiabdić analysed the factors that influence the adherence to low-calorie diets in overweight and obese patients and underlined how poor educational level is one of the negative predicting factors for a successful program [20]. Khan investigated the main factors of non-compliance in a population of diabetic patients and identified illiteracy as one of the most relevant negative prognostic factors [21]. Ferranti studied pregnant women with a history of gestational diabetes and underlined how patients with a higher education level and self-efficacy were those who followed a mostly adequate diet [22]. The educational level of the family is also important: several studies report an association between high educational level of the parents and quality of their children’s diet, both in early age and during adolescence [23].

The literature also shows suggests that, beyond education, the socio-economic level is related to the choice of “good quality” food [24]. Recently, Van Lenthe indicated that socio-economic inequalities in the choice of healthy food could be explained by differences in the levels of need fulfilment. By dividing people’s needs into five categories, according to the Maslow pyramid, the author showed an association between healthy food consumption and self-fulfilment, a category that encompassed the people with a higher educational level [25].

Several diseases are influenced by the socio-economic status: this is the also case of type 2 diabetes, which shares some features with chronic kidney disease, accounting for diet and need for self-management. Walker has recently analysed the association between socio-economic factors, psychological status and disease, highlighting a significant correlation between glycated haemoglobin, education, income, and self-efficacy [26].

Shah compared the treatment burden in celiac patients and in other chronic diseases, including CKD on dialysis. Celiac disease shares with CKD the importance of dietary compliance, which is made more complicated by the need to pay attention to gluten contamination; an issue not shared by CKD patients, who may, on the contrary, profit of occasional unrestricted meals. Also, poor diet compliance in patients with celiac disease has been associated with income (cost of food), lifestyle, educational level and time available to prepare meals [27].

Illiteracy and low socio-economic background have many further correlates, including poor oral status; indeed, our patient presented with chewing difficulties, a significant issue as for malnutrition, especially in the elderly, that should also be taken into account when prescribing a diet [28, 29].

Despite the initial difficulties, the daughter’s idea to convert the dietary advice into visual form allowed the patient to follow the diet in an optimal way. This also helped to avoid the risk of undernutrition, or of an unbalanced and over restricted protein intake, which could potentially be more deleterious than a high protein intake, both with respect to general health and to residual kidney function (Table 1).

As shown in the figures, using the symbols of a clock, the sun and the moon with the stars, the woman could understand what meal the pictures referred to. The patient knew that pasta, rice and bread had to be replaced by protein-free food in order to reduce total protein intake and to reach the target, calculated by her residual kidney function.

This self-made method achieved the goal of adequate compliance, reducing the patient’s “performance stress” and allowing her to follow the diet without depending on her family, who, due to work commitments and personal needs could not provide continuous assistance. In this process, we believe that the use of visual aids also played a fundamental role in reassuring the patient, who was scared with the prospect of starting dialysis primarily because of her family loss. We assume this psychological aspect was important since the patient continued to rely on her support, having learned how to follow her diet correctly.

This strategy permitted the stabilization of the residual kidney function, thereby fulfilling the patient’s wish to delay dialysis as much as possible.

## Conclusions

Our report warns against the discrimination of patients who are illiterate with regard to the prescription of low protein diets and the belief that they have limited understanding and poor compliance. The case described here may highlight how breaking cultural barriers can be empowering and enhance compliance and motivation, which may conversely be strengthened by the clinical success obtained. It also suggests the importance of the family support unit and underlines how CKD involves the whole family, and how family involvement may also be a resource for attaining compliance.

## Abbreviations

ACEi: Angiotensin converting enzyme inhibitors
ARBs: Angiotensin receptor blockers
CKD: Chronic kidney disease
CKD-EPI: Chronic Kidney Disease – Epidemiology Collaboration
eGFR: estimated glomerular filtration rate
